# Species Diversity and Distribution of Amphibians in Tangjiahe National Nature Reserve, China

**DOI:** 10.3390/biology14060614

**Published:** 2025-05-27

**Authors:** Mingfu Li, Mei Xiao, Li Zhao, Yiming Wu, Long Jin, Chengzhi Yan, Wenbo Liao

**Affiliations:** 1 Tangjiahe National Nature Reserve, Qingchuan 637007, China; limingfu@126.com (M.L.); xiaomei@126.com (M.X.); 2 Key Laboratory of Southwest China Wildlife Resources Conservation (Ministry of Education), China West Normal University, Nanchong 637009, China; lizhao@126.com (L.Z.); yimingwu@126.com (Y.W.); longjin07@126.com (L.J.); chengzhiyan@126.com (C.Y.); 3 Key Laboratory of Artificial Propagation and Utilization in Anurans of Nanchong City, China West Normal University, Nanchong 637009, China; 4 Key Laboratory of Ecological Adaptation in Amphibian in Sichuan Province, China West Normal University, Nanchong 637009, China

**Keywords:** amphibians, altitude, habitats, Tangjiahe National Nature Reserve, species composition

## Abstract

Mountain ecosystems offer valuable opportunities to study species distribution and diversity along altitudinal gradients, particularly for amphibians. This research examined amphibian species distribution, diversity, and conservation across an elevational gradient in the Tangjiahe National Nature Reserve, part of the Hengduan Mountains in southwestern China. A total of 25 amphibian species, encompassing 8 families and 2 orders, were documented, including three newly recorded species: *Fejervarya kawamurai*, *Polypedates braueri*, and *Boulenophrys minor*. Among these, eight species were designated as “threatened” under IUCN criteria and are listed on China’s Biodiversity Red List. Caijiaba exhibited the highest species diversity, whereas Shuichiping had the lowest. Fourteen species were found in terrestrial-farmland and aquatic-lotic habitats, which supported the greatest species richness. The distribution pattern along the altitudinal gradient showed peaks in species richness at 900–1100 m and 1900–2100 m elevation bands, with higher elevations displaying reduced richness. These findings highlighted the spatial characteristics of amphibian distribution and diversity across altitudinal ranges in the Tangjiahe National Nature Reserve and provide insights for formulating conservation policies and adaptive habitat management strategies.

## Simple Summary

Understanding amphibian distribution and diversity along altitudinal gradients is crucial for developing effective conservation policies. This study investigated species distribution and diversity of amphibians in the Tangjiahe National Nature Reserve, located in western China. We identified 25 amphibian species across 8 families and 2 orders, including three newly recorded species: *Fejervarya kawamurai*, *Polypedates braueri*, and *Boulenophrys minor*. We found that eight species were classified as “threatened” species according to International Union for Conservation of Nature (IUCN) criteria, and fourteen species inhabited terrestrial-farmland and aquatic-lotic environments. Species diversity analysis revealed the highest diversity in Caijiaba and the lowest in Shuichiping. Species richness peaked at elevation bands of 900–1100 m and 1900–2100 m, while elevations above 2300 m exhibited the lowest richness.

## 1. Introduction

Understanding the spatial patterns of biodiversity along geographical gradients is a fundamental question in ecology and conservation biology [1,2,3,4,5,6]. Mountain systems harbor a unique and substantial portion of global biodiversity [7,8,9]. Evidence suggests that environmental conditions associated with mountains influence biodiversity and species distribution across various animal groups [10,11,12,13,14]. Environmental conditions vary markedly over short spatial scales along elevational gradients in mountain systems, leading to diverse habitats and climatic zones within limited geographical distances [15]. Therefore, elevational gradients provide valuable opportunities to assess species distribution and diversity patterns [16].

Colder and less productive environments are typically associated with increasing elevation [17]. As a result, altitudinal variation in species richness often shows a decreasing trend with elevation or a peak at mid-elevations [18]. The decline in species richness at higher altitudes is likely attributable to harsher environmental conditions, including lower temperatures, reduced food availability, and decreased habitat complexity [19]. Amphibians represent an ideal model for investigating spatial patterns of species distribution due to their unique life cycle, which often involves both aquatic larval and terrestrial adult stages, making them highly sensitive to environmental disturbances [20,21]. Consequently, amphibian populations are vulnerable to various threats, including habitat degradation, pollution, and climate change [22,23]. As intermediate consumers, amphibians play a critical ecological role as both predators and prey, thereby contributing significantly to biodiversity and ecosystem stability [24]. Therefore, studying amphibian species distribution and conservation status is essential for comprehending evolutionary processes, ecological dynamics, and informing effective ecosystem management and conservation strategies.

Despite the global decline in amphibian populations, biodiversity hotspots continue to support amphibians that exhibit remarkable diversity and resilience [25,26]. the Tangjiahe National Nature Reserve represents a notable example of such a hotspot [27]. This reserve not only provides critical habitat for flagship species such as the giant panda, but also sustains a rich and diverse amphibian community [28]. Therefore, the reserve offers an optimal location for studying amphibian diversity and adaptability. Systematic investigations of amphibians in the Tangjiahe National Nature Reserve dates back to the 1980s. Between 1984 and 1996, a total of 150 amphibian specimens were collected, representing 18 species across 7 families and 2 orders [29]. Building on this baseline, further systematic surveys conducted in 2003 and 2013 confirmed the presence of 22 species spanning 8 families within the same orders [30]. Over time, the reserve has undergone substantial environmental changes, including strengthened conservation efforts, human resettlement, alterations in habitat structure, and the influence of climate variability [31,32]. As a result, earlier data are now inadequate to capture current patterns of amphibian diversity. An updated survey is urgently needed to assess recent changes in amphibian diversity and population dynamics over the past decade.

This study aims to assess amphibian diversity at five sites and analyze variations in species richness along an altitudinal gradient within the Tangjiahe National Nature Reserve. We first quantified amphibian species richness and diversity across these sites. Next, we compiled an updated inventory of amphibian species and identified potential threats to their persistence. Finally, we evaluated how species richness changes with increasing elevation. The results provide critical data and insights to inform regional ecological management and conservation strategies.

## 2. Materials and Methods

### 2.1. Study Area

This study was conducted in the Tangjiahe National Nature Reserve (32°30′–32°41′ N, 104°36′–104°56′ E), located in the northern part of Sichuan Province, China. The reserve borders Gansu Province and is located within the Minshan Mountain range. Established in 1978, it spans approximately 40,000 hectares and serves as a critical sanctuary for biodiversity conservation. The reserve is renowned for its rich ecosystems, ranging from subtropical forests to alpine meadows, with elevations between 1150 and 3830 m. It is part of the global biodiversity hotspot and a key component of the Giant Panda Habitat UNESCO World Heritage Site. Tangjiahe primarily protects species such as the giant panda, Sichuan takin, golden snub-nosed monkey, and clouded leopard. Over 3700 plant and animal species thrive here, including rare birds like the Chinese monal pheasant. As a transitional zone between the Qinghai-Tibet Plateau and the Sichuan Basin, the reserve plays a key role in climate regulation and ecological connectivity. The reported experiments complied with the current laws of China concerning animal experimentation, and ethical approval for specimen collection was received from the Ethical Committee for Animal Experiments at China West Normal University (Nanchong, China) (CWNU2024D012).

### 2.2. Sampling Collection

We conducted a comprehensive survey of amphibian species along a continuous 1800-meter elevational gradient, ranging from 800 m to 2600 m above sea level, within the Tangjiahe National Nature Reserve (Figure S1). This gradient encompassed a variety of terrestrial and aquatic habitats, facilitating a detailed analysis of species composition and elevational distribution. During the breeding season from May to August 2024, we implemented 39 line transects, each ranging from 200 to 500 m in length and 5 m in width. These transects were strategically positioned to cover diverse habitat types along the elevational gradient. Surveys were conducted nocturnally on rain-free nights by a team of five trained observers, who systematically searched for adult amphibians using handheld electric torches at a consistent pace of approximately 2.0 km/h. The locations of all observed individuals were recorded using the Beidou Navigation Satellite System, ensuring accurate and precise geospatial data collection.

### 2.3. Amphibian Conservation Status

The International Union for Conservation of Nature (IUCN) is a membership Union uniquely composed of both government and civil society organisations (https://www.iucnredlist.org/; accessed on 15 November 2024). The IUCN is the global authority on the status of the natural world and the measures needed to safeguard it. The conservation status of species can be estimated by the IUCN Red List of Threatened Species. Hence, we evaluated the conservation status of amphibian species in the Reserve by referring to both the IUCN Red List and the Red List of China’s Biodiversity. The IUCN Red List includes the following categories: Critically Endangered (CR), Endangered (EN), Vulnerable (VU), Near Threatened (NT), Least Concern (LC), Data Deficient (DD), and Not Evaluated (NE). The Red List of China’s Biodiversity includes Critically Endangered (CR), Endangered (EN), Vulnerable (VU), and Least Concern (LC).

### 2.4. Species Diversity 

The biodiversity of the amphibians was quantified using the Shannon–Wiener index (H′), calculated as H′ = −Σ(pi * ln(pi)), where pi denotes the proportion of individuals belonging to the i-th species relative to the total number of individuals observed. Community evenness of amphibians was assessed using Pielou’s index (E), calculated as E = H′/ln(S), where H′ is the Shannon–Wiener diversity index and S represents the total number of species in the amphibian community, reflecting how evenly individuals are distributed among the taxa. In this formula, E values closer to 1 indicate a higher level of evenness. The dominance of key species in the amphibian community was evaluated using the ecological dominance index (D), calculated as D = Σ(pi)^2^. In this formula, a D value closer to 1 indicates that the community is highly dominated by a few key species, while lower values suggest a more even distribution of species. This index is widely used to assess the extent to which dominant species control the structure of ecological communities. Amphibian species richness was quantified using Margalef’s index (R), defined as R = (S − 1)/ln(N), where S is the total number of species and N represents the total number of individuals sampled for altitudinal bands, providing a measure of diversity adjusted for sampling effort.

### 2.5. Statistical Analysis

All analyses were conducted on log-transformed data (base 10) using R version 4.0.1. We applied the Mann–Kendall trend analysis to examine potential associations between amphibian species count and collection year. Additionally, we utilized the Kruskal–Wallis test to assess species diversity across sites. Amphibians were distributed between 800 and 2500 m above sea level; therefore, this elevation range was divided into eight intervals at 200-m increments, and species richness within each interval was calculated. Consequently, we used linear regression analyses to explore the relationship between species richness and elevation. All probabilities were two-tailed, and significance was considered when *p* ≤ 0.05. Data are presented as means ± standard deviation (SD).

## 3. Results

### 3.1. Species Composition Variation

A total of 25 amphibian species were documented in the Tangjiahe National Nature Reserve, distributed across 8 families and 2 orders. The order Anura included 21 species, and the order Caudata included 4 species (Table S1). Of these species, 20 were captured during the field surveys, and 5 were recorded based on literature references (Table S1). A comparison of biodiversity surveys conducted between 1999 and 2013 in the Tangjiahe National Nature Reserve revealed the highest number of amphibian species recorded in 2024. Newly documented species included *Fejervarya kawamurai*, *Polypedates braueri*, and *Boulenophrys minor* (Table S1). The Mann–Kendall trend analysis indicated that the number of amphibian species tended to increase with the collection year (Figure S2; S = 5, Z = 1.44, *p* = 0.148).

### 3.2. Conservation Status of Amphibian Species

**Figure 1:**
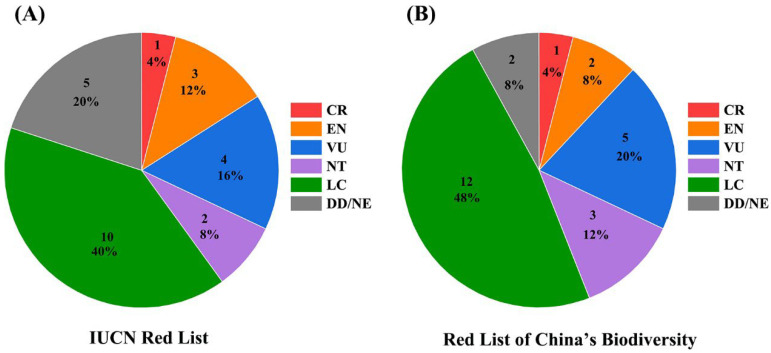
Conservation status of 25 amphibian species in Tangjiahe National Natural Reserve, western China. (**A**) IUCN criteria; (**B**) Red List of China’s Biodiversity.

The conservation status of the 25 amphibian species documented in the Tangjiahe National Nature Reserve was assessed using both the IUCN Red List and the Red List of China’s Vertebrates (Table S1). According to the IUCN Red List, one species (4%) is classified as Critically Endangered (CR), three species (12%) as Endangered (EN), and four species (16%) as Vulnerable (VU), collectively comprising 32% of the recorded species. These are categorized as “threatened species” under IUCN criteria. Additionally, two species (8%) are categorized as Near Threatened (NT), ten species (40%) as Least Concern (LC), while five species (20%) are classified as either Data Deficient (DD) or Not Evaluated (NE) (Figure 1A). Assessments based on the Red List of China’s Vertebrates yielded similar results, with one species (4%) classified as CR, two species (8%) as EN, and five species (20%) as VU, also accounting for 32% of the total species. Moreover, three species (12%) are identified as NT, while 12 species (48%) were categorized as LC (Figure 1B). Compared to the IUCN Red List, the Red List of China’s Vertebrates provides a more region-specific perspective, reflecting the unique ecological characteristics and conservation challenges faced by these species within China. The giant salamander (*Andrias davidianus*), one of the species classified, is consistently assessed as Critically Endangered under both the IUCN Red List and the Red List of China’s Vertebrates. Furthermore, five species (*A. davidianus*, *Batrachuperus pinchonii*, *B. tibetanus*, *Tylototriton wenxianensis*, and *Scutiger pingwuensis*) from this survey were listed as Class II nationally protected wildlife according to the List of State Key Protected Wild Animals in China. Of these species, *A. davidianus* is also included in Supplementary Materials of the Convention on International Trade in Endangered Species of Wild Fauna and Flora (CITES), while *T. wenxianensis* is listed in Supplementary Materials.

### 3.3. Amphibian Distribution Across Habitat Types

Distinct patterns of amphibian species distribution across habitat types were documented within the Tangjiahe National Nature Reserve (Table 1). Terrestrial-farmland and aquatic-lotic habitats supported the highest species richness, each harboring 14 species. Following these, aquatic-lentic habitats hosted 13 species, terrestrial-fossorial habitats accommodated 10 species, and terrestrial-highland habitats were home to 5 species. The remaining species were distributed across aquatic-rheophilic habitats, with one species occupying an arboreal environment. Notable variation in habitat breadth was observed among species. *Bufo gargarizans* demonstrated remarkable environmental adaptability, occupying both terrestrial and aquatic niches, a trait likely contributes to its widespread distribution and ecological success. In contrast, some species exhibited specialized habitat requirements. For instance, *Batrachuperus pinchonii* was exclusively found in aquatic-lotic environments, indicating a strong dependency on specific environmental conditions.

### 3.4. Species Diversity Across Collected Sites

Among the five study sites, Caijiaba exhibited the highest species diversity, whereas Shuichiping demonstrated the lowest (Table 2). Additionally, Shuichiping had the highest evenness index, and Baiguoping the lowest (Table 2). Species diversity and evenness indices did not differ significantly among the five sites (Diversity: Kruskal–Wallis test: H = 1.242, *p* = 0.336; Evenness: Kruskal–Wallis test: H = 1.012, *p* = 0.651). The ecological dominance index indicated no significant variation in ecological dominance among the study sites (Kruskal–Wallis test: H = 0.189, *p* = 0.912).

### 3.5. Species Richness Across Elevational Bands

**Figure 2:**
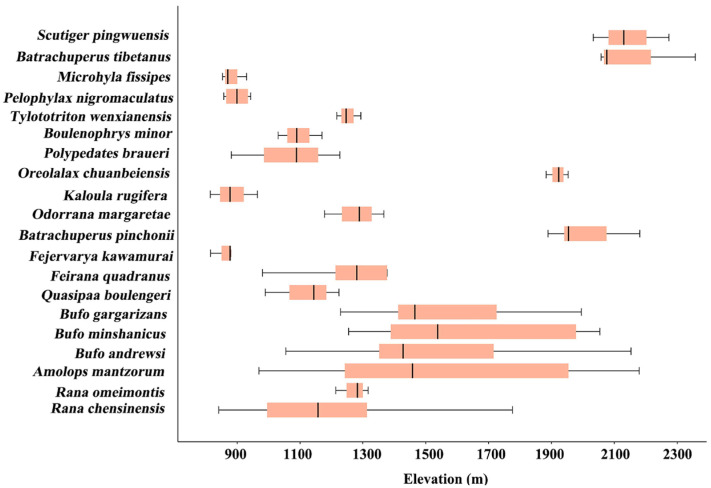
Elevational ranges for each amphibian species recorded in the Tangjiahe National Nature Reserve, western China.

Species distribution patterns along the altitudinal gradient revealed that the highest species richness was observed in the 900–1100 m and 1900–2100 m elevation bands (Figure S3). At elevations above 2300 m, only *Scutiger pingwuensis* and *Batrachuperus tibetanus* were recorded, representing the lowest species richness (Figure 2). Ordinary least squares regression analysis revealed that species richness did not increase with increasing altitude across elevation ranges (*R*^2^ = 0.114, *p* = 0.412). Additionally, significant differences in altitudinal ranges were observed at the species level (Figure 2). *Amolops mantzorum* and *Bufo andrewsi* exhibited broad altitudinal ranges, spanning from low to high elevations, reflecting greater ecological adaptability. In contrast, *Tylototriton wenxianensis* and *Rana omeimontis* were restricted to narrower elevation ranges, indicating stronger dependence on specific altitude conditions.

## 4. Discussion

Understanding species distribution and identifying threatened taxa and their conservation status are essential for guiding biodiversity conservation strategies in natural reserves. In this study, we conducted amphibian diversity surveys in the Tangjiahe National Nature Reserve since 1999, documenting species composition, distribution patterns, and conservation statuses, as well as analyzing diversity across habitat types and altitudinal gradients. Although recognized as a biodiversity hotspot, the reserve still harbors amphibian species with restricted ranges and specialized habitat requirements, posing substantial conservation challenges. Our findings provided updated data that enhance understanding of amphibian diversity and provided a robust foundation for refining conservation strategies and management efforts.

### 4.1. Species Composition

Amphibians are widely recognized as critical indicators of ecosystem health and biodiversity due to their pivotal role in trophic networks and heightened sensitivity to environmental changes [33,34]. Fluctuations in amphibian populations not only directly reflect the quality of ecological environments within protected areas but also serve as valuable references for informing conservation strategies and scientific decision-making. This study represents the fourth amphibian diversity survey conducted in the Tangjiahe National Nature Reserve over the past 40 years. Twenty-five species were recorded, representing the highest documented species richness in comparison to prior surveys of this reserve. We also found three species newly recorded during this time. These findings stand in contrast to the widespread decline of amphibian populations globally, which has been driven by threats such as habitat loss, environmental contamination, and climate change [26,35,36]. The observed increase in amphibian species number within the Tangjiahe National Nature Reserve may reflect the tangible ecological benefits of long-term conservation measures, including improved habitat connectivity and decreased human disturbances [37,38]. Nevertheless, *Megophrys omeimontis* and *Oreolalax nanjiangensis*, documented in earlier surveys, were absent during this field study. This absence may be explained by a combination of factors, including natural fluctuations in their population sizes, behavioral shifts that make them harder to detect, or temporary habitat changes influenced by localized climate variability. Therefore, long-term and systematic monitoring across different seasons and environmental conditions is essential to obtain a more comprehensive understanding of species diversity dynamics within protected areas.

### 4.2. Conservation Status of Amphibian Species

Amphibians, critical to ecosystem health, face unprecedented declines due to habitat loss, pollution, climate change, and disease. We evaluated the conservation status of 25 amphibian species in the Tangjiahe Nature Reserve and determined that 32% are classified as “threatened” (Critically Endangered (CR), Endangered (EN), and Vulnerable (VU)). Among these, *A. davidianus* is the sole species classified as Critically Endangered, as per both the IUCN Red List and the Red List of China’s Biodiversity [39]. This species is also designated as Class II protection under national regulations and is listed in Supplementary Materials of CITES, underscoring its critical conservation status and the urgent need for protective measures. As the largest amphibian globally, *A. davidianus* possesses significant ecological value but has experienced severe population declines due to habitat destruction and overexploitation for consumption [40,41]. Its presence within the reserve indicates suitable habitat conditions; however, factors such as habitat fragmentation, localized water quality issues, and human activities necessitate ongoing monitoring to assess their potential impacts on population recovery [42]. In addition to *A. davidianus*, four other amphibian species are designated as Class II protected under national regulations, with *Tylototriton wenxianensis* also listed in Supplementary Materials of CITES. *T. wenxianensis* has suffered significant habitat fragmentation, leading to a population decline exceeding 30% over the past decade [30]. The primary drivers of this decline include habitat degradation resulting from agricultural expansion, pesticide application, and fertilizer use [43,44]. Hence, we need to prioritize the protection and restoration of wetlands, forests, and freshwater ecosystems through legally enforced reserves and corridors. Limit deforestation, urban sprawl, and agricultural encroachment in critical habitats. Also, we need to educate communities on amphibians’ ecological roles via citizen science programs (e.g., frog monitoring) and school curricula. Moreover, we consider monitoring and mitigating outbreaks of deadly pathogens like chytrid fungus (*Batrachochytrium dendrobatidis*) through biosecurity protocols, captive breeding, and research focused on disease-resistant populations.

### 4.3. Amphibian Distribution Across Habitat Types

Habitat availability is a critical determinant of amphibian survival, directly influencing species persistence and ecological dynamics. According to the Global Amphibian Assessment, habitat loss and degradation represent major threats to approximately 63% of all amphibian species globally [45,46]. In this study, we demonstrated that aquatic-lotic and aquatic-lentic habitats supported the highest amphibian diversity, harboring 14 and 13 species, respectively. This finding suggests that these environments offer favorable habitat conditions and abundant resources, supporting species richness. Terrestrial-farmland and terrestrial-fossorial habitats were also relatively biodiverse, sustaining 14 and 10 species. These habitats collectively provide favorable microenvironmental conditions, including abundant food resources, stable microclimates, diverse breeding sites, and adequate refuges, which together promote high species richness [47,48,49]. Conversely, terrestrial-highland, aquatic-rheophilic, and arboreal environments supported markedly fewer species. These habitats were characterized by harsher environmental conditions, such as high altitude, extreme temperature fluctuations, or limited spatial coverage [50,51]. In addition to differences in species richness, we observed significant interspecific variation in habitat utilization. For example, *Bufo gargarizans* exhibited exceptional ecological flexibility, inhabiting aquatic habitats during the breeding season and transitioning to terrestrial habitats outside of the breeding period [27]. In contrast, certain species displayed more specialized habitat preferences. Notably, *Batrachuperus pinchonii* was exclusively observed in aquatic-lotic habitats. These findings underscore the importance of conserving diverse habitat types, particularly for species with specialized requirements. Such habitat specificity highlights the conservation significance of maintaining ecological integrity across diverse microhabitats within the reserve to preserve amphibian biodiversity. Future conservation strategies should prioritize habitat-specific management approaches to address the complex and heterogeneous needs of amphibian populations.

### 4.4. Species Diversity Across Collection Sites

Analyses of the Shannon–Wiener diversity index, Pielou’s evenness index, and ecological dominance index indicate that habitat fragmentation significantly reduces species diversity. Caijiaba exhibits high amphibian diversity with a relatively uniform species density distribution. Dominated by mixed forests, meadows, and perennial streams, Caijiaba maintains ecological integrity due to minimal human encroachment, supporting amphibian populations through two key mechanisms: (1) arthropod prey availability sustains their trophic requirements, and (2) ecotones between croplands and natural vegetation (e.g., grassy buffers and irrigation ditches) provide critical microhabitats for shelter and oviposition. Furthermore, community engagement initiatives led by Caijiaba authorities have mitigated anthropogenic pressures, enhancing habitat suitability for amphibians.

### 4.5. Species Richness Across Elevations

Amphibians inhabiting higher elevations encounter more severe environmental conditions compared to those at lower altitudes, leading to reduced species diversity [2]. Previous studies have indicated that mid-altitude areas exhibit the highest species richness and diversity along mountain gradients, as these zones offer more suitable habitats, greater resource availability, and reduced human disturbance [7]. In this study, we observed peak species richness within the 900–1100 m and 1900–2100 m elevation bands, while the lowest species richness was recorded above 2300 m. This pattern may be attributed to the diminished resource availability and suitable habitats at higher elevations, where harsher environmental conditions limit species survival. Conversely, the 900–1100 m and 1900–2100 m bands provide more favorable ecological conditions for amphibian persistence. Additionally, we noted relatively low species richness within the 1500–1700 m band, suggesting that human activities, such as tourism, at this elevation may intensify environmental filtering, leading to decreased species richness. Therefore, reducing human interference in this elevation range is essential for conserving amphibian diversity. Furthermore, variations in species’ altitudinal ranges highlight the relationship between elevation and amphibian diversity in the Tangjiahe National Nature Reserve, influencing both species richness and distribution patterns.

## 5. Conclusions

This preliminary investigation delineated the species diversity and distribution patterns of amphibians in the Tangjiahe National Nature Reserve. The findings revealed a low proportion of threatened species within the region, coupled with large population sizes. These results highlight that two decades of ecosystem conservation efforts in the Tangjiahe National Nature Reserve have effectively enhanced amphibian biodiversity, as evidenced by elevated species richness and diversity indices. Such progress is attributable to the implementation of targeted wildlife protection policies and adaptive habitat management strategies within the reserve. These findings provide a critical scientific foundation for targeted conservation strategies aimed at protecting local amphibian communities.

## Figures and Tables

**Table 1:** Amphibian species distribution from different habitats in the Tangjiahe National Natural Reserve, western China.

Habitat Types	Habitat Type							Species
	Arbo-Real	Terrestrial-Soil Cave	Terrestrial-Farmland	Terrestrial-Highland	Aquatic-Turbulence	Aquatic-Running Water	Aquatic-Quite Water	
5		√	√	√		√	√	*Bufo gargarizans*
5		√	√	√		√	√	*Bufo andrewsi*
5		√	√	√		√	√	*Bufo minshanicus*
2			√				√	*Fejervarya kawamurai*
2			√				√	*Rana chensinensis*
3		√	√				√	*Pelophylax nigromaculatus*
2			√				√	*Rana omeimontis*
1					√			*Amolops mantzorum*
1					√			*Amolops lifanensis*
2					√	√		*Odorrana margaretae*
3		√				√	√	*Quasipaa boulengeri*
3			√			√	√	*Feirana quadranus*
2		√	√					*Kaloula rugifera*
4	√	√	√				√	*Polypedates braueri*
2			√				√	*Hylarana guentheri*
2		√				√		*Boulenophrys minor*
2			√				√	*Microhyla fissipes*
3		√		√		√		*Oreolalax chuanbeiensis*
1						√		*Oreolalax nanjiangensis*
3		√		√		√		*Scutiger pingwuensis*
2			√			√		*Megophrys omeimontis*
2			√				√	*Tylototriton wenxianensis*
1						√		*Batrachuperus pinchonii*
1						√		*Batrachuperus tibetanus*
1						√		*Andrias davidianus*

√ indicates that a species has been found in this type of habitat.

**Table 2:** Amphibian diversity indices in the five sites of the Tangjiahe National Natural Reserve, western China.

Sites	Shannon–Wiener Diversity Index (H’)	Pielou’s Evenness Index (E)	Ecological Dominance Index (D)
Baiguoping	1.69	0.71	0.22
Baixiongping	1.69	0.87	0.23
Caijiaba	1.76	0.85	0.22
Motianling	1.67	0.86	0.23
Shuichiping	1.64	0.92	0.23

## Data Availability

The data presented in this study are available on request from the corresponding author.

## Supplementary Materials

The following supporting information can be downloaded at https://www.mdpi.com/article/10.3390/biology14060614/s1, Table S1: Amphibian species recorded in the Tangjiahe National Nature Reserve, China, including conservation status and habitat types; Figure S1: Topographic map showing study sites and line transects of amphibians in the Tangjiahe National Nature Reserve, China; Figure S2: Variation in number of amphibian species in the Tangjiahe National Nature Reserve from 1999, 2003, 2013, to 2024. Figure S3. Margalef richness index (R) of amphibians change with elevation variation in Tangjiahe National Natural Reserve, western China.

## References

[1] Gaston K.J. (2000). Global patterns in biodiversity. Nature.

[2] Wang X.Y., Zhong M.J., Zhang J., Xi X.F., Yang S.N., Jiang J.P., Hu J.H. (2022). Multidimensional amphibian diversity and community structure along a 2 600 m elevational gradient on the eastern margin of the Qinghai-Tibetan Plateau. Zool. Res..

[3] Shao W.J., Song X.Q., Chen C., Zhao L., Jin L., Liao W.B. (2022). Diversity and Distribution Pattern of Amphibians and Reptiles in Yingjing Area of the Giant Panda National Park. Chin. J. Zool..

[4] Yu F., Zhang L.J., Wang Y., Yi X.F., Zhang S., Ma J.M., Dong Z.M., Chen G.W., Ma K.M. (2023). High rodent abundance increases seed removal but decreases scatter-hoarding and seedling recruitment along an elevational gradient. Integr. Zool..

[5] Voelker G., Wogan G.O., Huntley J.W., Kaliba P.M., De Swardt D.H., Bowie R.C. (2024). Climate cycling did not affect haplotype distribution in an abundant Southern African avian habitat generalist species, the familiar chat (*Oenanthe familiaris*). Integr. Zool..

[6] Fernández-Cabello I., Franch M., Vilella M., Fernandez-Arrieta N., Rota M., Sanglas A., Baqué-Díaz E., Gallardet M., Federico P., Peris A. (2024). Assessing the role of habitat, climate, and anthropization gradients on terrestrial mammal diversity in the western Mediterranean basin. Integr. Zool..

[7] Rahbek C., Borregaard M.K., Colwell R.K., Dalsgaard B., Holt B.G., Morueta-Holme N., Nogues-Bravo D., Whittaker R.J., Fjeldsa J. (2019). Humboldt’s enigma: What causes global patterns of mountain biodiversity?. Science.

[8] Zhao X.B., Che X.L., Ning T., Zou F.S. (2023). Distribution of Birds in the High-Altitude Area of Mount Everest. Integr. Zool..

[9] Pan T., Zhang C.W., Terwengel P.O., Wang H., Ding L., Yang L.Y., Hu C.C., Li W.A., Zhou W.L., Wu X.B. (2024). Comparative phylogeography reveals dissimilar genetic differentiation patterns in two sympatric amphibian species. Integr. Zool..

[10] Feoktistova N.Y., Meschersky I.G., Shenbrot G.I., Puzachenko A.Y., Meschersky S.I., Bogomolov P.L., Surov A.V. (2023). Phylogeography of the Common Hamster (*Cricetus cricetus*): Paleoclimatic Reconstructions of Late Pleistocene Colonization. Integr. Zool..

[11] Lei B.Y., Zheng Z.F., Cui J.F., Zhao J., Newman C., Zhou Y.B. (2023). Ecotourist trail-use affects the taxonomic, functional and phylogenetic diversity of mammals in a protected area: Lessons for conservation management. Integr. Zool..

[12] Liu S.Q., Li K.X., Zheng Y.X., Xue J.Y., Wang S., Li S., Cao P., Liu F., Dai Q.Y., Feng X.T. (2024). Mitogenomes of museum specimens provide new insight into species classification and recently reduced diversity of highly endangered *Nomascus gibbons*. Integr. Zool..

[13] Allegrini C., Korine C., Krasnov B.R. (2024). Climatic gradients and forest composition shape bat communities in Eastern Mediterranean pine plantations. Integr. Zool..

[14] Hernández M., Hereira-Pacheco S., Alberdi A., de la Vega-pérez A.H., Estrada-Torres A., Ancona S., Navarro-Noya Y. (2024). DNA metabarcoding reveals seasonal changes in diet composition across four arthropod-eating lizard species (*Phrynosomatidae: Sceloporus*). Integr. Zool..

[15] Perrigo A., Hoorn C., Antonelli A. (2020). Why mountains matter for biodiversity. J. Biogeog..

[16] McCain C.M., Grytnes J.A. (2010). Elevational Gradients in Species Richness. Encyclopedia of Life Sciences (ELS).

[17] Körner C. (2000). Why are there global gradients in species richness? Mountains might hold the answer. Trends Ecol. Evol..

[18] Peters M.K., Hemp A., Appelhans T., Behler C., Classen A., Detsch F., Ensslin A., Ferger W.S., Frederiksen B.S., Gebert F. (2016). Predictors of elevational biodiversity gradients change from single taxa to the multi-taxa community level. Nat. Commun..

[19] Chamberlain D., Brambilla M., Caprio E., Pedrini P., Rolando A. (2016). Alpine bird distributions along elevation gradients: The consistency of climate and habitat effects across geographic regions. Oecologia.

[20] Wells K.D. (2007). The Ecology and Behavior of Amphibians.

[21] Coates M.I., Ruta M., Friedman M. (2008). Ever since owen: Changing perspectives on the early evolution of tetrapods. Annu. Rev. Ecol. Evol. Syst..

[22] Green D.M., Lannoo M.J., Lesbarrères D., Muths E. (2020). Amphibian population declines: 30 years of progress in confronting a complex problem. Herpetologica.

[23] Tan W.C., Herrel A., Rödder D. (2023). A global analysis of habitat fragmentation research in reptiles and amphibians: What have we done so far?. Biodivers. Conserv..

[24] Hocking D.J., Babbitt K.J. (2014). Amphibian contributions to ecosystem services. Herpetol. Conserv. Biol..

[25] Xu W., Wu Y.H., Zhou W.W., Chen H.M., Zhang B.L., Chen J.M., Xu W., Rao D.Q., Zhao H., Yan F. (2024). Hidden hotspots of amphibian biodiversity in China. Proc. Natl. Acad. Sci. USA.

[26] Campbell Grant E.H., Miller D.A.W., Muths E. (2020). A synthesis of evidence of drivers of amphibian declines. Herpetologica.

[27] Hu J.C. (2005). A Report of the Comprehensive Survey on Tangjiahe Nature Reserve in Sichuan.

[28] Wei J., Zheng W.C., Yang C., Zhang X.Y., Lan Y.X., Shen L.M., Hao J.F. (2019). Analysis on medicinal plant resources and diversity characteristics in the Tangjiahe National Nature Reserve. Acta Bot. Boreal. Occident. Sin..

[29] Shen L., Gao Z., Ou W., Chen W., Ma W. (1999). Survey Report on Amphibians and Reptiles in Tangjiahe Nature Reserve, Sichuan. Sichuan J. Zool..

[30] Zhang Z. (2016). Research on Biodiversity in Tangjiahe National Nature Reserve, China.

[31] Yao G., Fan Y., Li D., Hull V., Shen L., Li Y., Hu J. (2022). The influence of environmental variables on home range size and use in the golden snub-nosed monkey (*Rhinopithecus roxellana*) in Tangjiahe National Nature Reserve, China. Animals.

[32] He X., Wang X., Shen L., Zhuang Y. (2023). A Framework for Adaptive Collaborative Governance of National Park Communities and its Effectiveness in Practice: A Case Study of Community a in Tangjiahe Area of Giant Panda National Park. Landsc. Architect..

[33] Díaz-García J.M., Pineda E., López-Barrera F., Moreno C.E. (2017). Amphibian species and functional diversity as indicators of restoration success in tropical montane forest. Biodivers. Conserv..

[34] Sumanasekara V., Dissanayake D., Seneviratne H. Review on use of Amphibian Taxa as a Bio-Indicator for Watershed Health and Stresses. Proceedings of the NBRO Symposium Proceedings.

[35] Pigot A.L., Merow C., Wilson A., Trisos C.H. (2023). Abrupt expansion of climate change risks for species globally. Nat. Ecol. Evol..

[36] Luedtke J.A., Chanson J., Neam K., Hobin L., Maciel A.O., Catenazzi A., Borzee A., Hamidy A., Aowphol A., Jean A. (2023). Ongoing declines for the world’s amphibians in the face of emerging threats. Nature.

[37] Hughes A.R., Grabowski J.H., Leslie H.M., Scyphers S., Williams S.L. (2018). Inclusion of biodiversity in habitat restoration policy to facilitate ecosystem recovery. Conserv. Lett..

[38] De Zoysa M. (2022). Ecotourism development and biodiversity conservation in Sri Lanka: Objectives, conflicts and resolutions. Open J. Ecol..

[39] Cunningham A.A., Turvey S.T., Zhou F., Meredith H.M., Guan W., Liu X.L., Sun C.M., Wang Z.Q., Wu M.Y. (2016). Development of the Chinese Giant Salamander *Andrias davidianus* Farming Industry in Shaanxi Province, China: Conservation threats and opportunities. Oryx.

[40] Turvey S.T., Chen S., Tapley B., Wei G., Xie F., Yan F., Yang J., Liang Z., Tian H., Wu M. (2018). Imminent extinction in the wild of the world’s largest amphibian. Curr. Biol..

[41] Chai J., Lu C.Q., Yi M.R., Dai N.H., Weng X.D., Di M.X., Peng Y., Tang Y., Shan Q.H., Wang K. (2022). Discovery of a wild, genetically pure Chinese giant salamander creates new conservation opportunities. Zool. Res..

[42] Yan F., Lu J., Zhang B., Yuan Z., Zhao H., Huang S., Wei G., Mi X., Zou D., Xu W. (2018). The Chinese giant salamander exemplifies the hidden extinction of cryptic species. Curr. Biol..

[43] Ma Q., Wan L., Shi S., Wang Z. (2024). Impact of climate change on the distribution of three rare salamanders (*Liua shihi*, *Pseudohynobius jinfo* and *Tylototriton wenxianensis*) in Chongqing, China, and their conservation implications. Animals.

[44] Gong D., Mu M. (2008). Behavioral observations and descriptions of the endangered knobby newt *Tylototriton wenxianensis* and their application in conservation. Asian Herpetol. Res..

[45] Hoffmann M. (2008). Threatened Amphibians of the World.

[46] Ficetola G.F., Rondinini C., Bonardi A., Baisero D., Padoa-Schioppa E. (2015). Habitat availability for amphibians and extinction threat: A global analysis. Divers. Distrib..

[47] Burrow A., Maerz J. (2022). How plants affect amphibian populations. Biol. Rev..

[48] Moreira L.F.B., Maltchik L. (2014). Does organic agriculture benefit anuran diversity in rice fields?. Wetlands.

[49] Harper E.B. (2007). The Role of Terrrestrial Habitat in the Population Dynamics and Conservation of Pond-Breeding Amphibians.

[50] Khatiwada J.R., Zhao T., Chen Y., Wang B., Xie F., Cannatella D.C., Jiang J. (2019). Amphibian community structure along elevation gradients in eastern Nepal Himalaya. BMC Ecol..

[51] Smalling K.L., Rowe J.C., Pearl C.A., Iwanowicz L.R., Givens C.E., Anderson C.W., McCreary B., Adams M.J. (2021). Monitoring wetland water quality related to livestock grazing in amphibian habitats. Environ. Monit. Assess..

