# Supplementary material for: Species Diversity and Distribution of Amphibians in Tangjiahe National Nature Reserve, China
Source: Biology (Basel). 2025 May 27;14(6):614. doi: 10.3390/biology14060614 (PMC12189923; doi:10.3390/biology14060614)

Electronic Supplementary Material

Species Diversity and Distribution of Amphibians in Tangjiahe National Nature Reserve, China

Mingfu Li [1], Mei Xiao [1], Li Zhao [2,3,4], Yiming Wu [2,3,4], Long Jin [2,3,4], Chengzhi Yan [2,3,4] and Wenbo Liao [2,3,4,*]

[1] Tangjiahe National Nature Reserve, Qingchuan 637007, China; limingfu@126.com (M.L.); xiaomei@126.com (M.X.)
[2] Key Laboratory of Southwest China Wildlife Resources Conservation (Ministry of Education), China West Normal University, Nanchong 637009, China; lizhao@126.com (L.Z.); yimingwu@126.com (Y.W.); longjin07@126.com (L.J.); chengzhiyan@126.com (C.Y.)
[3] Key Laboratory of Artificial Propagation and Utilization in Anurans of Nanchong City, China West Normal University, Nanchong 637009, China
[4] Key Laboratory of Ecological Adaptation in Amphibian in Sichuan Province, China West Normal University, Nanchong 637009, China

.

Table S1 Amphibian species recorded in Tangjiahe National Nature Reserve, China, including conservation status and habitat types

Order	Family	Species	IUCN Red List	Red List of China's Biodiversity	List of Key Protected Wild Animals in China	CITES	Habitat Type
**Caudata**	Hynobiidae	*Batrachuperus pinchonii*	VU	VU	II		ALO
		Batrachuperus tibetanus	VU	VU	II		ALO
	Cryptobranchidae	*Andrias davidianus*	CR	CR	II	I	ALO
	Salamandridae	*Tylototriton wenxianensis*	VU	VU	II	II	ALE, TFA
**Anura**	Megophryidae	*Oreolalax chuanbeiensis*	EN	EN			ALO, TH, TFO
		Oreolalax nanjiangensis	VU	NT			ALO
		Scutiger pingwuensis	EN	EN	II		ALO, TH, TFO
		Boulenophrys minor	LC	LC			ALO, TFO
		Megophrys omeimontis	NT	VU			ALO, TFA
	Bufonidae	*Bufo gargarizans*	LC	LC			ALE, ALO, TH, TFA, TFO
		Bufo andrewsi	DD	DD			ALE, ALO, TH, TFA, TFO
		Bufo minshanicus	DD	DD			ALE, ALO, TH, TFA, TFO
	Ranidae	*Hylarana guentheri*	LC	LC			ALE, TFA
		Pelophylax nigromaculatus	NT	NT			ALE, TFA, TFO
		Odorrana margaretae	LC	LC			ALO, AR
		Rana omeimontis	LC	LC			ALE, TFA
		Rana chensinensis	LC	LC			ALE, TFA

		Amolops lifanensis	LC	LC			AR
		Amolops mantzorum	LC	LC			AR
	Microhylidae	*Microhyla fissipes*	LC	LC			ALE, TFA
		Kaloula rugifera	LC	LC			TFA, TFO
	Rhacophoridae	*Polypedates braueri*	NE	LC			ALE, TFA, TFO, AL
	Dicroglossidae	*Quasipaa boulengeri*	EN	VU			ALE, ALO, TFO
		Feirana quadranus	DD	NT			ALE, ALO, TFA,
		Fejervarya kawamurai	NE	LC			ALE, TFA

VU: critically endangered, EN: endangered, LC: least concern, NT: near threatened, VU: vulnerable, DD: data Deficient, NE: not evaluated; TFA: terrestrial-farmland, ALO: aquatic-lotic, ALE: aquatic-lentic, TFO: terrestrial-fossorial, TH: terrestrial-highland, AR: aquatic- rheophilic, AL: arborea.

Figure S1: Topographic map showing study sites and line transects of amphibians in Tangjiahe National Nature Reserve, China;

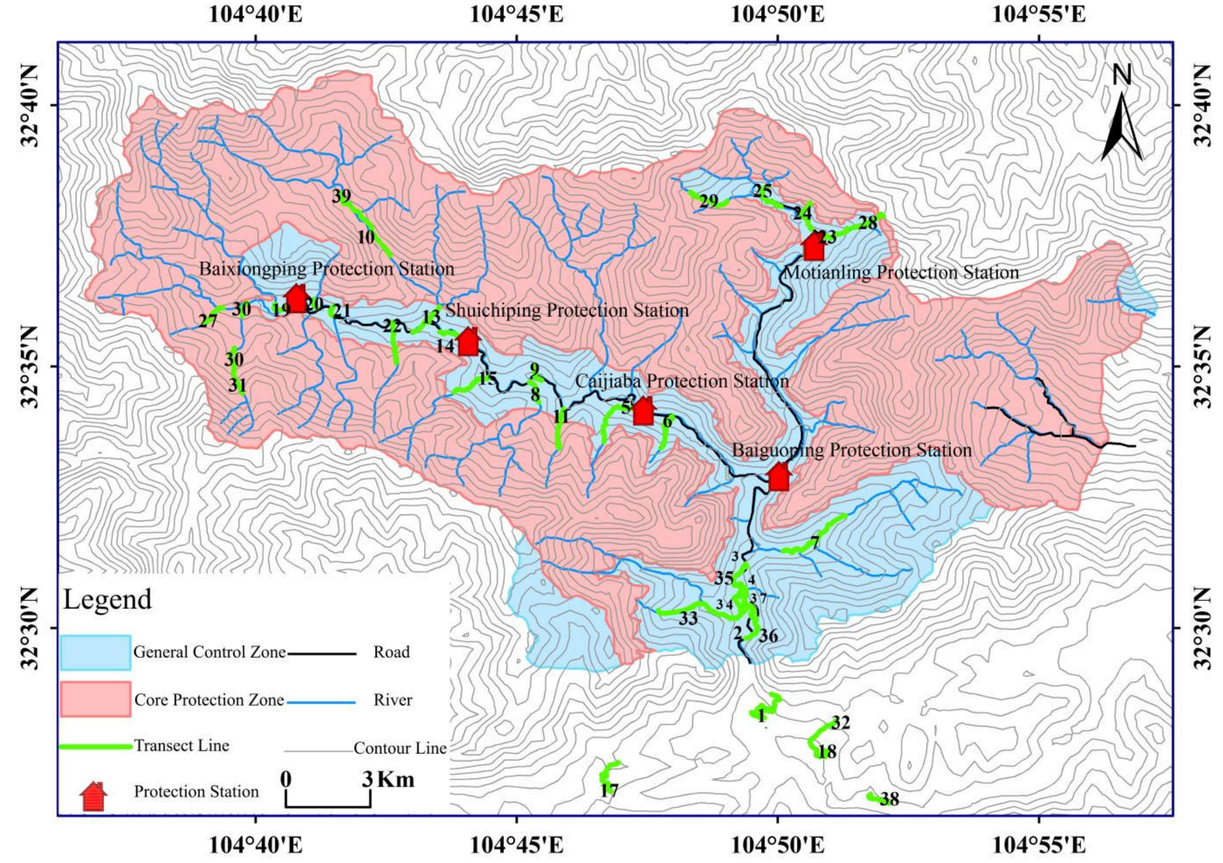

Figure S2: Variation in number of amphibian species in Tangjiahe National Nature Reserve between from 1999, 2003, 2013 to 2024.

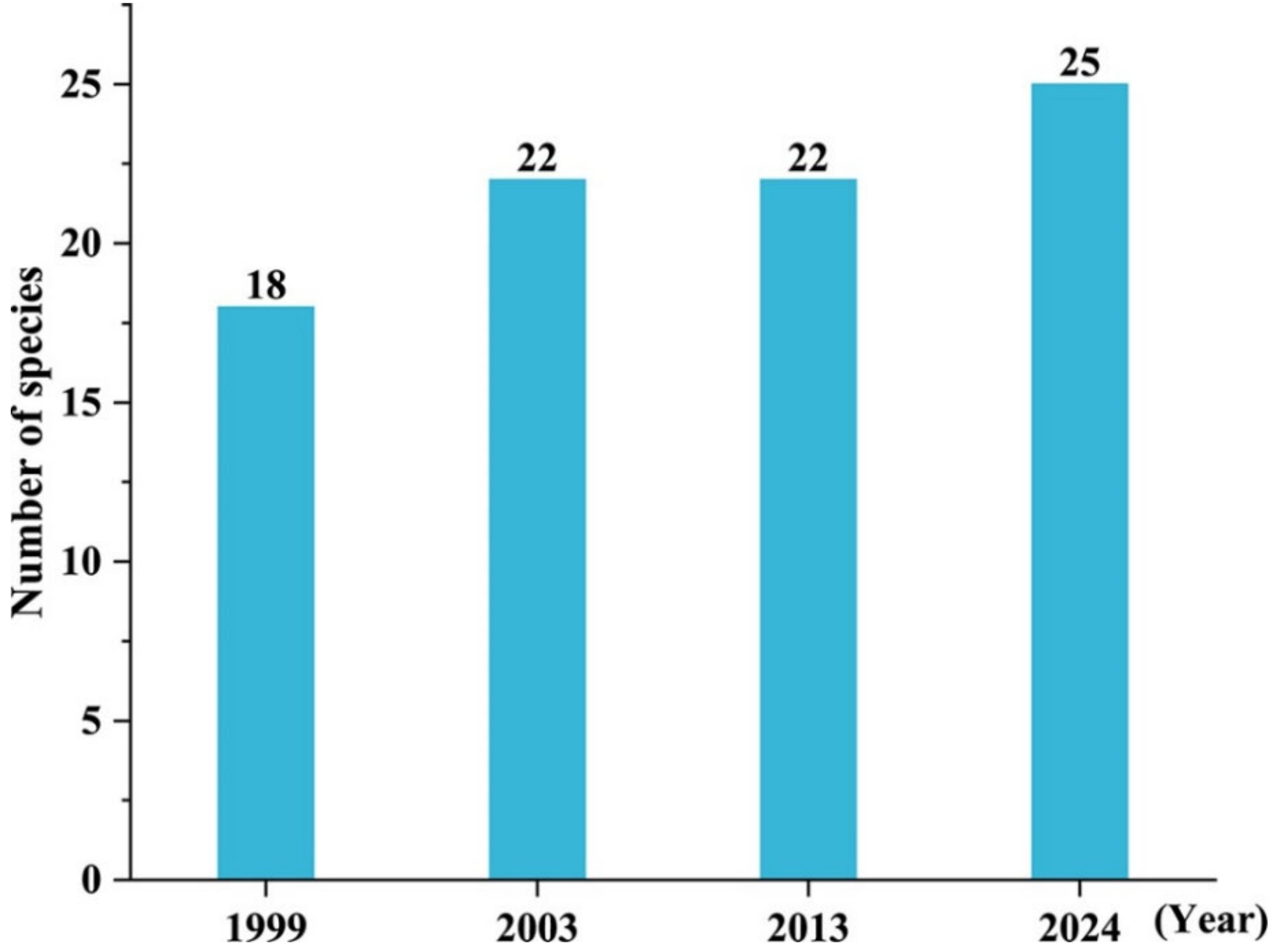

Figure S3. Margalef richness index (R) of amphibians change with elevation variation in Tangjiahe National Natural Reserve, western China.

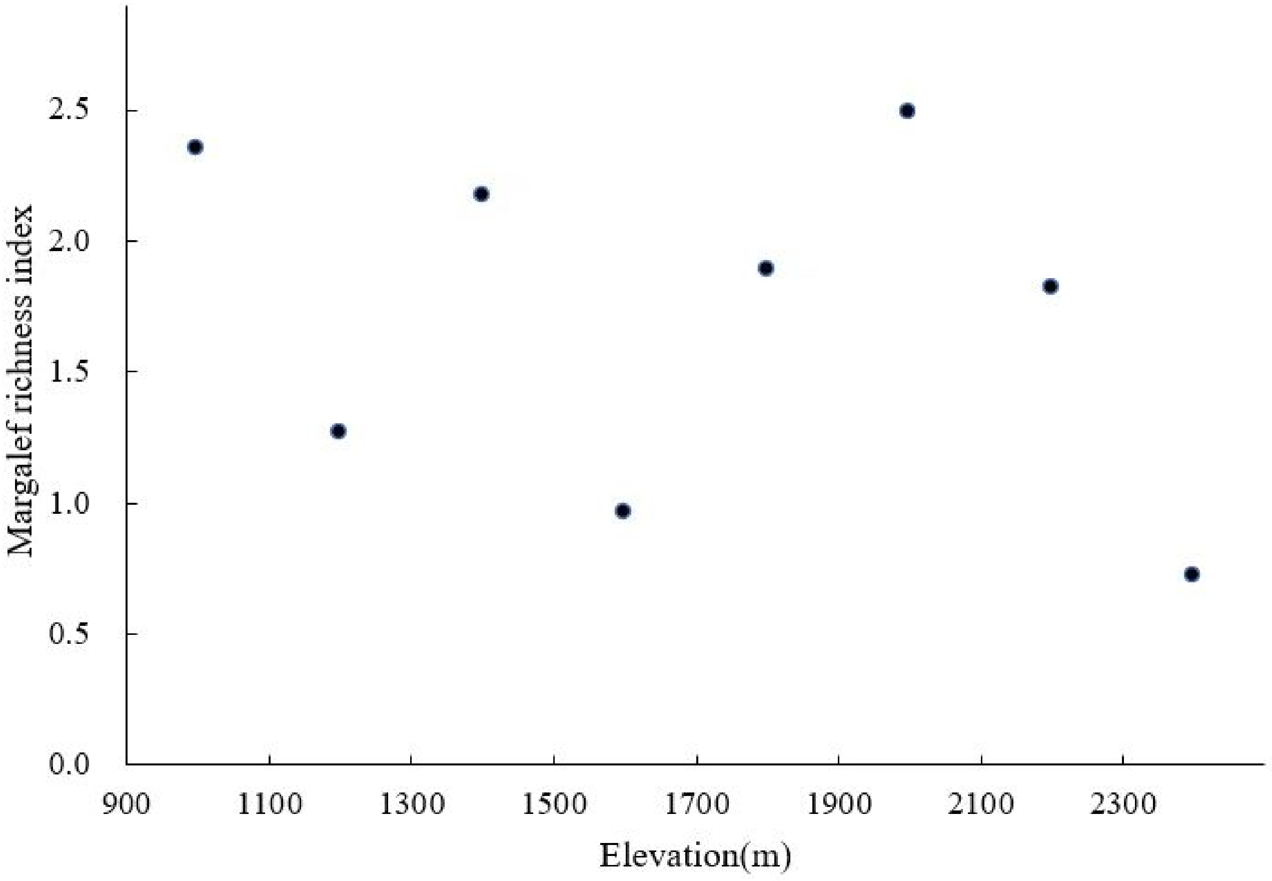

Supplement: Supplementary file 1 [file biology-14-00614-s001.zip › biology-3633953-supplementary.pdf]
